# Diagnosis- and Prognosis-Related Gene Alterations in *BCR::ABL1*-Negative Myeloproliferative Neoplasms

**DOI:** 10.3390/ijms241613008

**Published:** 2023-08-21

**Authors:** Soji Morishita, Norio Komatsu

**Affiliations:** 1 Development of Therapies against MPNs, Juntendo University Graduate School of Medicine, 2-1-1 Hongo, Bunkyo-ku, Tokyo 113-8421, Japan; 2 Advanced Hematology, Juntendo University Graduate School of Medicine, 2-1-1 Hongo, Bunkuo-ku, Tokyo 113-8421, Japan; 3 PharmaEssentia Japan, Akasaka Center Building 12 Fl, 1-3-13 Motoakasaka, Minato-ku, Tokyo 107-0051, Japan

**Keywords:** *BCR::ABL1*-negative myeloproliferative neoplasms, gene mutations, diagnostic marker, prognosis, genetic background

## Abstract

*BCR::ABL1*-negative myeloproliferative neoplasms (MPNs) are a group of hematopoietic malignancies in which somatic mutations are acquired in hematopoietic stem/progenitor cells, resulting in an abnormal increase in blood cells in peripheral blood and fibrosis in bone marrow. Mutations in *JAK2*, *MPL*, and *CALR* are frequently found in *BCR::ABL1*-negative MPNs, and detecting typical mutations in these three genes has become essential for the diagnosis of *BCR::ABL1*-negative MPNs. Furthermore, comprehensive gene mutation and expression analyses performed using massively parallel sequencing have identified gene mutations associated with the prognosis of *BCR::ABL1*-negative MPNs such as *ASXL1*, *EZH2*, *IDH1/2*, *SRSF2*, and *U2AF1*. Furthermore, single-cell analyses have partially elucidated the effect of the order of mutation acquisition on the phenotype of *BCR::ABL1*-negative MPNs and the mechanism of the pathogenesis of *BCR::ABL1*-negative MPNs. Recently, specific *CREB3L1* overexpression has been identified in megakaryocytes and platelets in *BCR::ABL1*-negative MPNs, which may be promising for the development of diagnostic applications. In this review, we describe the genetic mutations found in *BCR::ABL1*-negative MPNs, including the results of analyses conducted by our group.

## 1. Introduction

**Figure 1:**
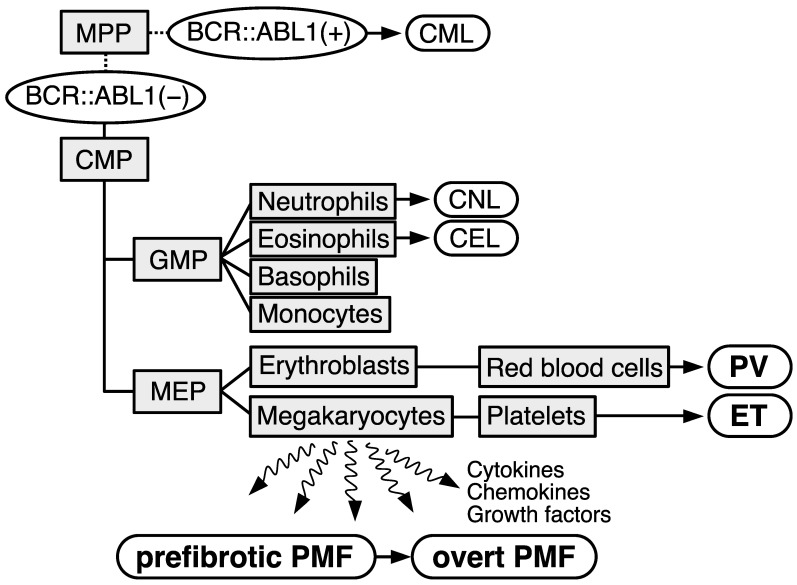
Schematical illustration describing the subtypes of myeloproliferative neoplasms (MPNs). Cell types are depicted as light gray squares. MPP: multipotent progenitor; CMP: common myeloid progenitor; GMP: granulocyte-monocyte progenitor; MEP: megakaryocyte–erythrocyte progenitor. Subtypes of MPNs are depicted as white rounded rectangles. Chronic myeloid leukemia (CML) exhibits *BCR::ABL1* gene (*BCR::ABL1*(+)). Other subtypes are stratified as *BCR::ABL1*-negative (−) MPNs. CNL: chronic neutrophilic leukemia; CEL: chronic eosinophilic leukemia; PV: polycythemia vera; ET: essential thrombocythemia; PMF: primary myelofibrosis.

**Figure 2:**
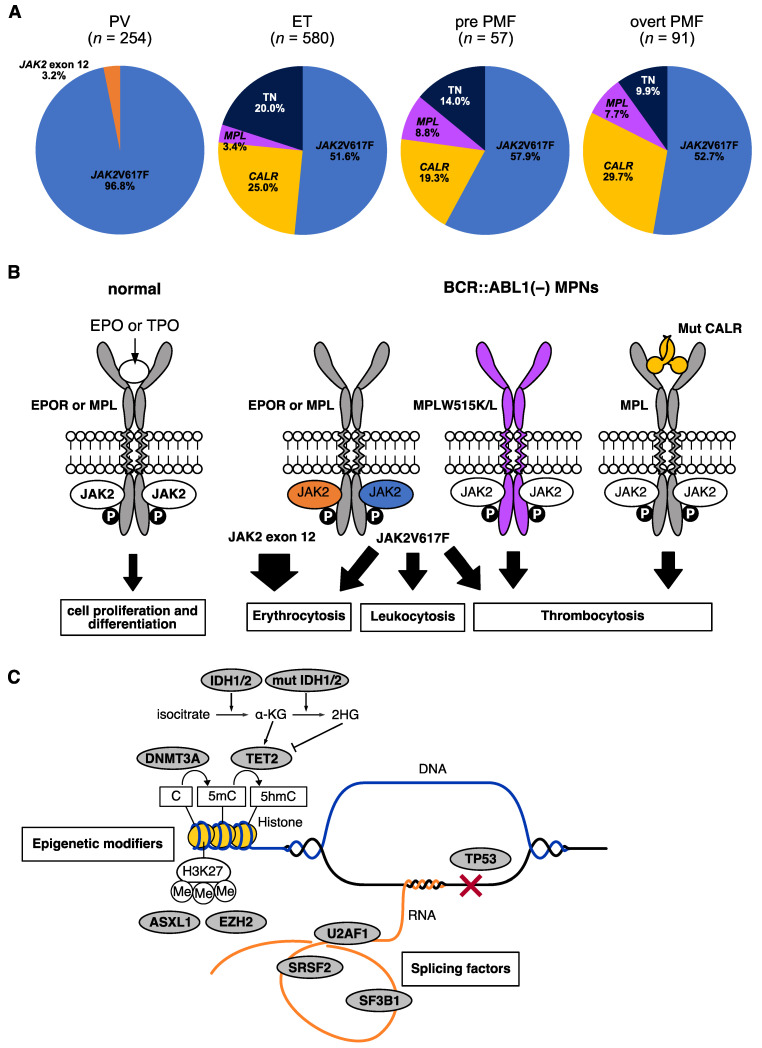
(**A**): Distribution of driver gene mutations for *BCR::ABL1*-negative MPNs in our Japanese cohort. Triple-negative: TN. (**B**): Driver gene mutations in *BCR::ABL1*-negative MPNs. Cell proliferation signal is regulated through the binding of cytokines (erythropoietin: EPO or thrombopoietin: TPO) to the receptors (erythropoietin receptor: EPOR or thrombopoietin receptor: MPL, left panel). However, in the driver gene-mutated *BCR::ABL1*-negative MPNs, downstream signal cascades constitutively activate owing to the mutant proteins without the binding of cytokines. JAK2 exon 12 mutations activate strong signals, especially for erythrocytosis, whereas JAK2V617F activates trilineage signals (erythrocytosis, leukocytosis, and thrombocytosis). MPLW515L/K and mutant CALR activate MPL signaling and trigger thrombocytosis (right panels). (**C**): Typical nondriver gene transcripts identified in *BCR::ABL1*-negative MPNs. Mutations that occur in these genes disrupt the gene regulations and my affect the prognosis of *BCR::ABL1*-negative MPNs. IDH1/2 generates a-ketoglutaric acids (a-KG) from isocitrate. Mutant IDH1/2 generates 2-hydroxyglutaric acid (2HG) from a-KG, resulting in the suppression of TET2. DNMT3A and TET2 act as DNA methylation and demethylation enzymes by methylating cytosine to 5-methylcytosine (5mC) and oxidizing 5mC to 5-hydroxymethylcytosine (5hmC), respectively. ASXL1 and EZH2 regulate the transcription through the trimethylation of histone 3 lysine 27. SF3B1, SRSF2, and U2AF1 function as splicing factors. TP53 is a well-known guardian gene of carcinogenesis.

Myeloproliferative neoplasms (MPNs) are characterized as a clonal proliferation of hematopoietic stem/progenitor cells, which cause an increase in one or more mature myeloid lineage cells. MPNs consist of multiple subgroups: chronic myeloid leukemia (CML); polycythemia vera (PV); essential thrombocythemia (ET); prefibrotic primary myelofibrosis (PMF); overt PMF; chronic neutrophilic leukemia (CNL); chronic eosinophilic leukemia (CEL); and unclassifiable MPN, not otherwise specified (Figure 1) [1,2]. In general, CML involves a typical driver gene alteration, the *BCR::ABL1* fusion gene, and more rare diseases, namely CNL, CEL, and unclassifiable MPN, not otherwise specified, have been treated as independent diseases. In contrast, patients with *BCR::ABL1*-negative MPNs (PV, ET, prefibrotic PMF, and overt PMF) transform to other subgroups with a 10–15% frequency and share common gene mutations, namely *JAK2* mutations (V617F and exon 12), *MPL*W515L/K, and *CALR* exon 9 frameshift mutations, in a mutually exclusive manner [3,4,5,6,7,8,9]. The frequencies of the three gene mutations in our cohort are shown in Figure 2A. Since these mutations exhibit oncogenic properties, they are defined as driver mutations of *BCR::ABL1*-negative MPNs (Figure 2B). Ahead of the *MPL* and *CALR* mutations, *JAK2* mutations were listed as one of the major criteria in the 2008 WHO classification [10]. After identifying *MPL* and *CALR* mutations, the three driver genes are now available for a revised 2022 WHO classification, which highlights the importance of genetic testing in *BCR::ABL1*-negative MPN, and have become essential for the definitive diagnosis of *BCR::ABL1*-negative MPNs [1,2]. However, a portion of patients with ET and PMFs (approximately 10–15%) exhibit negativity for all these driver mutations, which are considered to be triple-negative (TN). As for the diagnosis of TN, an exclusion of the possibility of nonneoplastic blood cell mass elevation should be carefully conducted in addition to a bone marrow (BM) biopsy which is mandatory to confirm that the histopathological characteristics of the BM morphology match the diagnostic criteria of *BCR::ABL1*-negative MPNs regardless of the presence of driver gene mutations, because no diagnostic markers have been identified [11]. To find novel gene alterations, genome-wide approaches targeting TN cases have been performed, and although several novel driver mutations in *BCR::ABL1*-negative MPNs have been found, the functional role of these mutations in the pathogenesis of *BCR::ABL1*-negative MPNs remains unclear [12]. Recently, *CREB3L1* overexpression in RNA from the platelets of MPN patients was identified, which may be a comprehensive diagnostic marker for *BCR::ABL1*-negative MPNs [13]. In addition, several mutations on the genes functioning as epigenetic modifiers and splicing factors were identified not only in leukemias/myelodysplastic syndromes but also in *BCR::ABL1*-negative MPNs, and the association of these mutations with the prognosis of *BCR::ABL1*-negative MPNs has been analyzed (Figure 2C) [14,15,16,17]. Furthermore, the influence of genetic background on the predisposition to *BCR::ABL1*-negative MPNs has been suggested in studies with large cohorts and familial *BCR::ABL1*-negative MPN pedigrees [18,19]. This article describes genetic abnormalities identified in *BCR::ABL1*-negative MPNs and their associations with the development or prognosis of *BCR::ABL1*-negative MPNs.

## 2. *JAK2* Mutations

**Figure 3:**
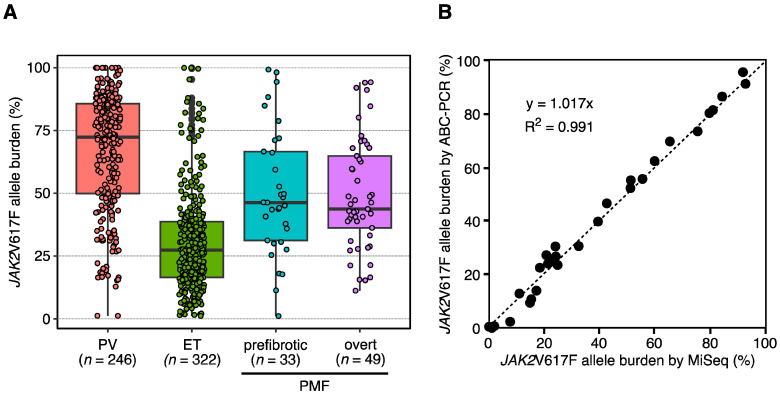
(**A**): Scatter plot showing the relationship between *JAK2*V617F allele burden measured using massively parallel sequencing (MiSeq, *x*-axis) and those measured using ABC-PCR (*y*-axis). The correlation coefficient of R^2^ was calculated as 0.991; (**B**): box plot showing the *JAK2*V617F allele burden among patients with PV (red), ET (green), prefibrotic PMF (blue), and overt PMF (purple) harboring the *JAK2*V617F mutation (>1.0%). The median/mean *JAK2*V617F allele burden is 72.3/66.8% in PV, 27.3/30.2% in ET, 46.3/50.2% in prefibrotic PMF, and 43.7/49.3% in overt PMF.

The *JAK2* mutations considered as driver mutations of *BCR::ABL1*-negative MPNs are V617F substitution and complex mutations, including missense and in-frame deletions/insertions at exon 12 [3,4,5,6]. These mutations concentrate around the JH2 domain, which suppresses the kinase activity of the JH1 domain in JAK2 under a static state. *JAK2* mutations decrease the suppression of kinase activity by the JH2 domain, resulting in the constitutive activation of JAK2. The *JAK2*V617F mutation is a single nucleotide alteration from guanine to thymine at nucleotide position 1849, which causes an amino acid change from V (valine, GTC) to F (phenylalanine, TTC) at codon 617. In addition, the *JAK2*V617F mutation was initially identified from the three driver gene mutations of *BCR::ABL1*-negative MPNs [3,4,5] and has been most frequently identified among the patients with *BCR::ABL1*-negative MPNs, and the positivity is approximately 97% in PV and approximately 50% in ET and PMF. In contrast, *JAK2* exon 12 mutations are specific for PV, with 3% positivity (Figure 1A), and a variety of mutations have been identified at *JAK2* exon 12 (Supplementary Table S1, according to the Catalogue Of Somatic Mutations In Cancer (COSMIC) database, as of June 2023) [20,21]. Clinically, patients with PV harboring the *JAK2*V617F mutation exhibit pancytosis, including leukocytosis, thrombocytosis, and erythrocytosis, whereas those harboring the *JAK2* exon 12 mutation show only an aggressive increase in red cell mass. As for the relationship between the prognosis and *JAK2* mutations, no significant differences between *JAK2*V617F-mutated and *JAK2* exon 12-mutated patients with PV have been observed [22]; nevertheless, *JAK2*V617F is a well-known risk factor of thrombosis [23,24]. Thrombosis promoted through the increased neutrophil extracellular trap formation was observed in the *JAK2*V617F-mutated murine model [25]. *JAK2*V617F is also useful for monitoring the efficacy of treatments or predicting the outcome of patients. Pegylated interferon-α, for example, is one of the recently developed drugs against MPNs and decreases the *JAK2*V617F allele burden in patients [26,27,28]. To date, pegylated interferon-α is the only agent that specifically affects the hematopoietic stem/progenitor cells of *BCR::ABL1*-negative MPNs, indicating the need for methodologies that quantify the mutant burden to monitor the efficacy of such agents. Therefore, a unique quantitative technique for assessing the *JAK2*V617F allele burden, alternately named binding-probe competitive PCR (ABC-PCR), has been developed [29]. The *JAK2*V617F mutant allele load (allele burden) in Japanese patients with *BCR::ABL1*-negative MPNs quantified by utilizing ABC-PCR showed the tendency of distribution of *JAK2*V617F allele burden between the subtypes of *BCR::ABL1*-negative MPNs (Figure 3A). Furthermore, ABC-PCR enables precise quantification of the *JAK2*V617F allele burden by correlating it with fluorescence intensity and may be as effective as massively parallel sequencing (MPS, Figure 3B). By utilizing ABC-PCR, the relationship between the allele burden and clinical significance was clarified; the increase in mutant burden during the follow-up is associated with the transformation to secondary myelofibrosis [30]. Furthermore, more studies have shown that thrombotic risks increase in patients bearing high allele burden [31] and more frequent cooccurrence of chronic kidney disease and tendency for disease progression [32], implicating the relationship between the clinical relevance and mutant allele burdens. The series of results obtained by groups studying Western patients and our group studying Japanese patients demonstrate that the quantification of the *JAK2*V617F allele burden may be used as an indicator of drug efficacy or to predict the adverse prognosis of *BCR::ABL1*-negative MPNs. Moreover, the highly sensitive detection of *JAK2*V617F is desired for the early diagnosis of MPNs. Melting curve analysis after T allele enrichment (MelCaTle) detects the *JAK2*V617F allele at a single-copy level by eliminating the *JAK2* wild-type allele using a peptide nucleic acid probe and *Bst*XI restriction enzyme [33]. The combination of ABC-PCR and MelCaTle enables precise detection of the *JAK2*V617F mutation at a single-molecule level and accurately quantify the *JAK2*V617F burden in patients with MPNs.

## 3. *MPL* Mutations

The majority of MPL mutations involve the substitution of W (tryptophane, c.1542-1544TGG) at codon 515 with other nucleic acids, causing W515L/K/A/R (leucine/lysine/alanine/arginine) mutations [7,34]. In addition to these W515 mutations, the substitution of S (serine) at codon 505 with N/C (asparagine/cysteine) has been identified [35,36]. These mutations are located at the membrane-spanning segment of MPL and are considered to involve conformation changes that trigger constitutive activation of the downstream molecules. Although the frequency of MPL mutations in *BCR::ABL1*-negative MPNs is low (5% at most in ET, <10% in PMF), considering that mutant CALR binds to MPL and activates downstream signals [37], signal activation through MPL may play a key role in the pathogenesis of *BCR::ABL1*-negative MPNs [38]. Patients harboring *MPL* mutations are also relatively rare, but a meta-analysis unifying seven studies clarified that patients with ET harboring an *MPL* mutation showed higher risks for thrombosis than those harboring a *JAK2*V617F mutation [39]. In line with this study, Japanese patients with ET harboring *MPL* mutations demonstrated a higher risk for thrombosis [40]. Therefore, *MPL* mutations may be one of the adverse factors of thrombosis.

## 4. *CALR* Exon 9 Frameshift Mutations

**Figure 4:**
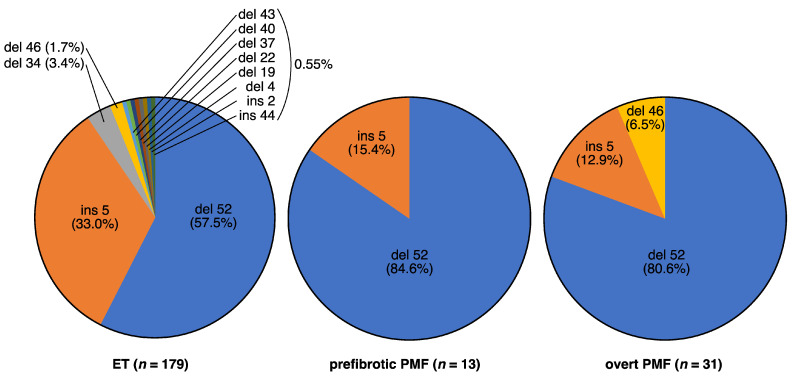
Distribution of *CALR* frameshift mutations analyzed in our data. ET (**left**, *n* = 179), prefibrotic PMF (**middle**, *n* = 13), and overt PMF (**right**, *n* = 31).

The *CALR* mutation is the most recently discovered among the driver gene mutations in *BCR::ABL1*-negative MPNs and is found in approximately 20–30% of patients with ET and PMF [8,9]. This mutation is characterized by the presence of a deletion or insertion at the end of exon 9, the final exon of the *CALR* gene. Over 100 variations have been found (Supplementary Tables S2 and S3, according to the COSMIC database, as of January 2023), all of which cause the same frameshift and produce a common amino acid sequence at the C-terminus when translated into protein. Among them, deletions of 52 bases (type 1, p.L367fs*46) and insertions of 5 bases (type 2, p.K385fs*47) are the major mutations, accounting for approximately 85% of the observed variations (Figure 4). Mutant CALR activates downstream signaling by forming homomultimeric complexes through a new amino acid sequence generated by the mutation, changing the structure of the CALR protein and allowing it to bind with MPL [37,41,42]. Patients with overt PMF harboring a *CALR* mutation have a better prognosis than those with other driver gene mutations [43]. Regarding the *CALR* mutations in overt PMF, *CALR* type 1 mutations are dominant, and patients harboring the type 2 mutation exhibit a poorer prognosis than those with the type 1 mutation [44,45].

## 5. Triple-Negative *BCR::ABL1*-Negative MPNs

A portion of patients with *BCR::ABL1*-negative MPNs (none or rare in PV, 10–15% in ET, and ~10% in PMF) have none of the driver mutations, referred to as TN cases. Noncanonical somatic mutations at driver genes of *BCR::ABL1*-negative MPNs (e.g., *JAK2*G571S and *MPL*S204F/P) have been identified in TN cases; however, it should be considered that these mutations do not account for all the remaining cases (Supplementary Table S4) [12,46] and no evidence of cytokine-independent cell growth has been reported for these mutations. Whole exome sequencing and analysis of TN-ET have shown that approximately half of the patients exhibited polyclonal cell differentiation, which implies that some cases of thrombocytosis in TN-ET may be caused by nonneoplastic diseases [12,47]. In addition, several cases of nonneoplastic erythrocytosis (NNE) showing low EPO levels (<4.2 IU/mL, low EPO NNE) were identified in our analysis. BM biopsy samples derived from low EPO NNE patients were diagnosed by histopathologists, resulting in the denial of PV in all cases [48]. Careful histopathological diagnosis of promising diagnostic markers for *BCR::ABL1*-negative MPNs is mandatory for such puzzling cases.

## 6. *CREB3L1* as a Novel Diagnostic Marker of *BCR::ABL1*-Negative MPNs

To diagnose TN cases in practice, a histopathological diagnosis of the BM biopsy is required. However, the discrimination of TN from reactive cases is challenging because the pathological diagnosis of *BCR::ABL1*-negative MPNs is not always reproducible, even for expert hematopathologists. By focusing on the fact that typical clinical presentations (i.e., thrombocytosis) of ET are similar regardless of the presence and type of driver gene mutations, and that the downstream RNA expression may be common and different from that in reactive cases, differential expression analysis utilizing RNA from platelet-rich plasma (PRP) obtained from ET and reactive thrombocytosis patients was conducted. As a result, *CREB3L1* was found to be specifically overexpressed among ET patients [13]. CREB3L1 is a transcription factor that localizes in the endoplasmic reticulum (ER), migrates into the nucleus in response to ER stress, and induces the expression of various genes [49]. Although the role of CREB3L1 in the pathogenesis of *BCR::ABL1*-negative MPNs remains unclear, the IRE1a/XBP1 pathway, which is an ER stress-responsible pathway other than CREB3L1, is activated by the CALR type 1 mutation and drives *BCR::ABL1*-negative MPNs [50]. In breast and bladder cancer, *CREB3L1* is highly methylated, and *CREB3L1* expression is inversely correlated with tumor grade, indicating that it acts in a tumor-suppressive manner [51,52].

**Figure 5:**
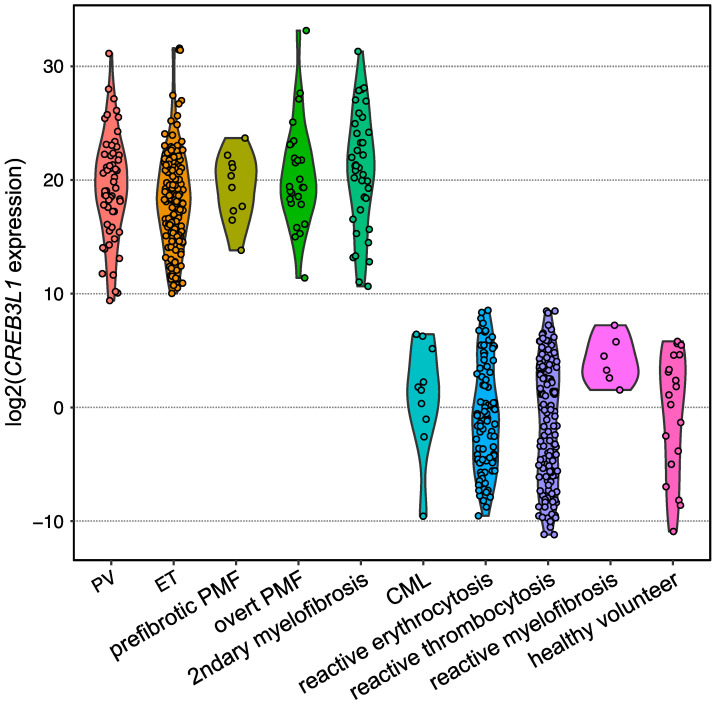
Violin plot showing the expression levels of *CREB3L1* measured using reverse transcription quantitative PCR. Dots represent the *CREB3L1* levels of the individuals, which are expressed as the value relative to the mean expression levels among healthy volunteers. *B2M* was used as an internal control.

Expansion of the testing of *CREB3L1* overexpression for other subtypes of *BCR::ABL1*-negative MPNs harboring one of the driver gene mutations in the validation analysis by employing quantitative PCR revealed that *CREB3L1* was overexpressed widely among *BCR::ABL1*-negative MPNs compared with those of reactive cases and healthy volunteers (Figure 5). The area under the ROC curve showed that the sensitivity and specificity were both 1.0000, indicating that the *CREB3L1* overexpression in PRP discriminates driver gene-mutated *BCR::ABL1*-negative MPNs from reactive cases [13]. Further investigations are required to determine whether *CREB3L1* expression is a diagnostic marker for *BCR::ABL1*-negative MPNs, including TN cases. In our cohort, 20 cases without any driver mutations were definitively diagnosed with TN-ET according to the pathological characteristics. Among them, eight cases did not express *CREB3L1* mRNA. Based on these findings, TN-ET cases were stratified into two groups (*CREB3L1*-positive TN-ET and *CREB3L1*-negative TN-ET) and the clinical parameters of the two groups were monitored. As a result, the platelet counts of two patients with *CREB3L1*-negative TN-ET decreased to normal levels during observation. In both cases, the BM biopsies at the time of initial diagnosis were consistent with ET, but the BM examinations performed at the time of spontaneous regression were negative for ET [13]. Therefore, aggressive treatments, such as anticancer drugs, should be avoided, and careful follow-up observation is recommended in cases with *CREB3L1*-negative TN-ET.

## 7. Nondriver Mutations and Their Association with the Prognosis of *BCR::ABL1*-Negative MPNs

Comprehensive genome analyses, such as MPS, have identified some mutations relating to epigenetic modification and RNA-splicing among patients with *BCR::ABL1*-negative MPNs at low frequency [14,15]. MPS-based comprehensive target resequencing methodology focusing on these nondriver mutations also revealed that the mutations highly accumulated in patients with *BCR::ABL1*-negative MPNs were correlated with a poor prognosis. Based on these findings, mutation-enhanced prognostic scoring systems based on the positivity of nondriver mutations have been proposed and are widely used to estimate the prognostic risks of patients with *BCR::ABL1*-negative MPNs. For example, the mutation-enhanced international prognostic scoring system (MIPSS) 70+ v2.0, which was originally designed for patients with PMF aged 70 years or younger and eligible for transplantation, uses the *CALR*, *ASXL1*, *EZH2*, *IDH1/2*, *SRSF2*, and *U2AF1*Q157 mutations [53]. MIPSS-ET and -PV are available for classifying the prognostic risks of patients with ET and PV, using the *SF3B1*, *SRSF2*, *TP53,* and *U2AF1* mutations for ET and the *SRSF2* mutation for PV [54].

Similar to the results obtained by other groups, patients with PMF harboring *ASXL1*, *EZH2*, and/or *SRSF2* mutations exhibited significantly shorter 5-year overall survival, and these gene mutations are also the poor prognostic factors of PMF that were demonstrated in the Japanese cohort [16]. Furthermore, regarding ET and PV, the frequencies of *ASXL1* and *EZH2* mutations increase as the diseases progresses from ET or PV to prefibrotic PMF and overt PMF, whereas the frequencies of *DNMT3A* and *TET2* mutations are unrelated to disease type. This implies that *ASXL1* and *EZH2* mutations are related to disease progression, whereas *DNMT3A* and *TET2* mutations may trigger the disease. Logistic regression analysis showed that *ASXL1* mutation-positive ET/PV patients had a high rate of progression to leukemia and myelofibrosis [17]. Nonetheless, the prognosis of *BCR::ABL1*-negative MPNs may be affected by the timing and order of the acquisition of these mutations. Although the effect of timing remains unclarified, the effect of acquisition order on the phenotype of *BCR::ABL1*-negative MPNs has been studied. Ortmann and colleagues cultured mononuclear cells isolated from the peripheral blood of *BCR::ABL1*-negative MPN patients who were positive for both *JAK2*V617F and *TET2* mutations in methylcellulose medium and examined the positivity of *JAK2* and *TET2* mutations in the BFU-E colonies. As a result, patients with *JAK2*V617F-first colonies had stronger clinical symptoms and were at higher risk of developing thrombosis and PV than those with the *TET2*-first colonies. The authors proposed a model in which the respective clinical symptoms differ according to the acquisition order of *JAK2* and *TET2* mutations [55]. Moreover, a colony assay targeting a patient with myelodysplastic syndrome (MDS)/MPN-RS-T harboring both *JAK2* exon 12 (p.H538_K539delinsL) and *SF3B1*E622D mutations demonstrated that *SF3B1*-mutated clones existed in isolation but all clones with *JAK2* exon 12 mutations had accompanying *SF3B1* mutations. This indicates that the *SF3B1* mutation triggers pancytopenia in the cells and then the *JAK2* mutation is acquired as a second-hit mutation, causing thrombocytosis in the patient to rescue the pancytopenia to some extent [56]. The above two studies show that the first-hit gene mutations characterize the basal phenotype of the disease, and the second-hit mutations contribute additional clinical presentations of the disease; therefore, the order in which the gene mutations are acquired may explain the development and progression of the disease. More recently, comprehensive RNA expression analysis with single-cell resolution has been employed to estimate cell profiling in patients. Tong and colleagues found prominent megakaryocyte lineage priming and elevated interferon signaling in hematopoietic stem cells (HSCs) in *JAK2*V617F-mutated ET patients, and the pathogenesis and therapeutic responses were dependent on the *JAK2*V617F heterogeneity of HSCs [57]. A more precise association between the disease development and traits, including single nucleotide polymorphisms (SNPs) or the differentiation and proliferation of neoplastic cells in *BCR::ABL1*-negative MPNs, may be revealed by novel approaches such as the network genome-wide association studies (networkGWAS) and RNA velocity-based algorithms (e.g., CellRank) [58,59,60].

## 8. Genetic Background Enhancing the Risk of Developing *BCR::ABL1*-Negative MPNs

The accumulation of mutations is highly dependent on age, and elderly individuals sometimes develop clonal hematopoiesis because of acquired mutations [61,62]. Such individuals are diagnosed with clonal hematopoiesis of intermediate potential (CHIP) or age-related clonal hematopoiesis (ARCH) [63,64]. Regarding the development of CHIP/ARCH, it has been experimentally demonstrated using the zebrafish model that mutant clones increase by developing clonal fitness, which is driven by enhanced resistance to inflammatory signals [65]. Notably, *JAK2*V617F has been identified with a frequency of approximately 0.1% in an analysis of 49,488 individuals, and 7 patients harboring *JAK2*V617F (48 individuals were removed for originally having MPNs) were then considered as CHIP/ARCH in the final cohort. Furthermore, the *JAK2*V617F mutant burden in CHIP/ARCH increases by 0.55% per year in the study, implying that the *JAK2*V617F-mutated cells acquire a mild growth advantage, and therefore the development of *BCR::ABL1*-negative MPNs may progress over time [66]. This implication is supported by another investigation, which clarified that *JAK2*V617F mutations occur decades before *BCR::ABL1*-negative MPN diagnosis, increase the fitness of HSCs, and induce a megakaryocyte–erythroid differentiation bias [67]. CHIP/ARCH individuals also have an increased risk of developing hematologic malignancies or cardiovascular diseases in patients with additional mutations [68].

Some studies have investigated the impact of genetic background on the risk of developing *BCR::ABL1*-negative MPNs. For example, the *JAK2* 46/1 haplotype concomitant with *JAK2*V617F, *RBBP6*, *SH2B3*, and *TERT* mutations was shown to increase the risk of developing *BCR::ABL1*-negative MPNs [69,70,71,72,73,74]. A GWAS analysis of 888,503 individuals, including 2949 patients with *BCR::ABL1*-negative MPNs, identified 17 loci, including *JAK2* and *TERT* [18]. SNPs located at the identified loci, such as rs17879961 (located at *CHEK2* exon 5) and rs534137 (located at the promoter region of *GFI1B*), were considered to induce the instability of HSCs homeostasis and may predispose patients to *BCR::ABL1*-negative MPNs. In addition, a germline frameshift mutation at *Carbohydrate Sulfotransferase 15 (CHST15)* has been identified among some familial *BCR::ABL1*-negative MPN pedigrees with the same geographical origin [19]. Mutant *CHST15* reduces expression levels of *CHST15* and its target genes, which induces a chronic inflammatory response, and this may also predispose patients to *BCR::ABL1*-negative MPNs. The above mutations may trigger genetic instability in the cells, induce chronic inflammation, and impose cell fitness toward a predisposition to *BCR::ABL1*-negative MPNs.

In addition to SNPs that confer higher susceptibility to develop *BCR::ABL1*-negative MPNs, SNPs that affect the phenotype, prognosis, and response to therapies of *BCR::ABL1*-negative MPNs have been reported and summarized [75]. Although allelic frequencies are not rare (0.114822 for rs6198, 0.279039 for rs1024611, and 0.444187 for rs2431697 by gnomAD, respectively), a poorer prognosis was observed among patients with PMF harboring both *JAK2*V617F and homozygous mutations of rs6198 locating at *NR3C1* than those bearing wild-type *NR3C1* [76], rs1024611 at *CCL2* strongly correlated to the *CCL2* expression and the myelofibrosis grade [77], and homozygous rs2431697 at miR-146a was associated with myelofibrosis progression [78]. Furthermore, *IL28B* rs12979860 homozygous phenotype showed hematologic response in patients with PV treated with interferon-α [79]. These SNPs would be useful to consider as a therapeutic strategy for *BCR::ABL1*-negative MPNs.

## 9. Conclusions

This review focused on the gene alterations involved in the pathogenesis or development of *BCR::ABL1*-negative MPNs. Notably, the number of mutations associated with *BCR::ABL1*-negative MPNs is smaller than those of acute leukemias and solid tumors, and the genetic mutation analyses play a significant role in confirming diagnosis and prognosis. On top of this, quantitation of mutant burden in the patients can provide important clinical information such as transformation to myelofibrosis and drug responses in the patients with *BCR::ABL1*-negative MPNs.

## 10. Future Perspective

The function of each mutation found in *BCR::ABL1*-negative MPNs is relatively easy to analyze; therefore, clarifying the pathogenesis of *BCR::ABL1*-negative MPNs may provide a model for other hematopoietic malignancies and solid cancers. The latest comprehensive gene expression technologies and subsequent statistical analyses help to elucidate the cell behavior with single-cell resolution. Moreover, deep machine learning approaches using artificial intelligence may assist in developing novel diagnostic/prognosis markers and help elucidate the pathogenesis of *BCR::ABL1*-negative MPNs, potentially leading to personalized therapies [80].

## Data Availability

No new data was created in this review article.

## Supplementary Materials

The following supporting information can be downloaded at: https://www.mdpi.com/article/10.3390/ijms241613008/s1.

## References

[1] Arber D.A., Orazi A., Hasserjian R.P., Borowitz M.J., Calvo K.R., Kvasnicka H.M., Wang S.A., Bagg A., Barbui T., Branford S. (2022). International Consensus Classification of Myeloid Neoplasms and Acute Leukemias: Integrating morphologic, clinical, and genomic data. Blood.

[2] Khoury J.D., Solary E., Abla O., Akkari Y., Alaggio R., Apperley J.F., Bejar R., Berti E., Busque L., Chan J.K.C. (2022). The 5th edition of the World Health Organization Classification of Haematolymphoid Tumours: Myeloid and Histiocytic/Dendritic Neoplasms. Leukemia.

[3] Kralovics R., Passamonti F., Buser A.S., Teo S.S., Tiedt R., Passweg J.R., Tichelli A., Cazzola M., Skoda R.C. (2005). A gain-of-function mutation of JAK2 in myeloproliferative disorders. N. Engl. J. Med..

[4] Baxter E.J., Scott L.M., Campbell P.J., East C., Fourouclas N., Swanton S., Vassiliou G.S., Bench A.J., Boyd E.M., Curtin N. (2005). Acquired mutation of the tyrosine kinase JAK2 in human myeloproliferative disorders. Lancet.

[5] James C., Ugo V., Le Couedic J.P., Staerk J., Delhommeau F., Lacout C., Garcon L., Raslova H., Berger R., Bennaceur-Griscelli A. (2005). A unique clonal JAK2 mutation leading to constitutive signalling causes polycythaemia vera. Nature.

[6] Scott L.M., Tong W., Levine R.L., Scott M.A., Beer P.A., Stratton M.R., Futreal P.A., Erber W.N., McMullin M.F., Harrison C.N. (2007). JAK2 exon 12 mutations in polycythemia vera and idiopathic erythrocytosis. N. Engl. J. Med..

[7] Pardanani A.D., Levine R.L., Lasho T., Pikman Y., Mesa R.A., Wadleigh M., Steensma D.P., Elliott M.A., Wolanskyj A.P., Hogan W.J. (2006). MPL515 mutations in myeloproliferative and other myeloid disorders: A study of 1182 patients. Blood.

[8] Nangalia J., Massie C.E., Baxter E.J., Nice F.L., Gundem G., Wedge D.C., Avezov E., Li J., Kollmann K., Kent D.G. (2013). Somatic CALR mutations in myeloproliferative neoplasms with nonmutated JAK2. N. Engl. J. Med..

[9] Klampfl T., Gisslinger H., Harutyunyan A.S., Nivarthi H., Rumi E., Milosevic J.D., Them N.C., Berg T., Gisslinger B., Pietra D. (2013). Somatic mutations of calreticulin in myeloproliferative neoplasms. N. Engl. J. Med..

[10] Vardiman J.W., Thiele J., Arber D.A., Brunning R.D., Borowitz M.J., Porwit A., Harris N.L., Le Beau M.M., Hellstrom-Lindberg E., Tefferi A. (2009). The 2008 revision of the World Health Organization (WHO) classification of myeloid neoplasms and acute leukemia: Rationale and important changes. Blood.

[11] Barbui T., Thiele J., Gisslinger H., Kvasnicka H.M., Vannucchi A.M., Guglielmelli P., Orazi A., Tefferi A. (2018). The 2016 WHO classification and diagnostic criteria for myeloproliferative neoplasms: Document summary and in-depth discussion. Blood Cancer J..

[12] Milosevic Feenstra J.D., Nivarthi H., Gisslinger H., Leroy E., Rumi E., Chachoua I., Bagienski K., Kubesova B., Pietra D., Gisslinger B. (2016). Whole-exome sequencing identifies novel MPL and JAK2 mutations in triple-negative myeloproliferative neoplasms. Blood.

[13] Morishita S., Yasuda H., Yamawaki S., Kawaji H., Itoh M., Edahiro Y., Imai M., Kogo Y., Tsuneda S., Ohsaka A. (2021). CREB3L1 overexpression as a potential diagnostic marker of Philadelphia chromosome-negative myeloproliferative neoplasms. Cancer Sci..

[14] Tefferi A., Lasho T.L., Finke C.M., Elala Y., Hanson C.A., Ketterling R.P., Gangat N., Pardanani A. (2016). Targeted deep sequencing in primary myelofibrosis. Blood Adv..

[15] Tefferi A., Lasho T.L., Guglielmelli P., Finke C.M., Rotunno G., Elala Y., Pacilli A., Hanson C.A., Pancrazzi A., Ketterling R.P. (2016). Targeted deep sequencing in polycythemia vera and essential thrombocythemia. Blood Adv..

[16] Morishita S., Ochiai T., Misawa K., Osaga S., Inano T., Fukuda Y., Edahiro Y., Ohsaka A., Araki M., Komatsu N. (2021). Clinical impacts of the mutational spectrum in Japanese patients with primary myelofibrosis. Int. J. Hematol..

[17] Morishita S., Hashimoto Y., Furuya C., Edahiro Y., Ochiai T., Shirane S., Inano T., Yasuda H., Ando M., Araki M. (2023). Non-driver gene mutation analysis in a large cohort of polycythemia vera and essential thrombocythemia. Eur. J. Haematol..

[18] Bao E.L., Nandakumar S.K., Liao X., Bick A.G., Karjalainen J., Tabaka M., Gan O.I., Havulinna A.S., Kiiskinen T.T.J., Lareau C.A. (2020). Inherited myeloproliferative neoplasm risk affects haematopoietic stem cells. Nature.

[19] Chen Y., Zhang Y., Wang Z., Wang Y., Luo Y., Sun N., Zheng S., Yan W., Xiao X., Liu S. (2022). CHST15 gene germline mutation is associated with the development of familial myeloproliferative neoplasms and higher transformation risk. Cell Death Dis..

[20] Percy M.J., Scott L.M., Erber W.N., Harrison C.N., Reilly J.T., Jones F.G., Green A.R., McMullin M.F. (2007). The frequency of JAK2 exon 12 mutations in idiopathic erythrocytosis patients with low serum erythropoietin levels. Haematologica.

[21] Scott L.M., Beer P.A., Bench A.J., Erber W.N., Green A.R. (2007). Prevalance of JAK2 V617F and exon 12 mutations in polycythaemia vera. Br. J. Haematol..

[22] Tondeur S., Paul F., Riou J., Mansier O., Ranta D., Le Clech L., Lippert E., Tavitian S., Chaoui D., Mercier M. (2020). Long-term follow-up of JAK2 exon 12 polycythemia vera: A French Intergroup of Myeloproliferative Neoplasms (FIM) study. Leukemia.

[23] Barbui T., Finazzi G., Carobbio A., Thiele J., Passamonti F., Rumi E., Ruggeri M., Rodeghiero F., Randi M.L., Bertozzi I. (2012). Development and validation of an International Prognostic Score of thrombosis in World Health Organization-essential thrombocythemia (IPSET-thrombosis). Blood.

[24] Barbui T., Vannucchi A.M., Buxhofer-Ausch V., De Stefano V., Betti S., Rambaldi A., Rumi E., Ruggeri M., Rodeghiero F., Randi M.L. (2015). Practice-relevant revision of IPSET-thrombosis based on 1019 patients with WHO-defined essential thrombocythemia. Blood Cancer J..

[25] Wolach O., Sellar R.S., Martinod K., Cherpokova D., McConkey M., Chappell R.J., Silver A.J., Adams D., Castellano C.A., Schneider R.K. (2018). Increased neutrophil extracellular trap formation promotes thrombosis in myeloproliferative neoplasms. Sci. Transl. Med..

[26] Verstovsek S., Komatsu N., Gill H., Jin J., Lee S.E., Hou H.A., Sato T., Qin A., Urbanski R., Shih W. (2022). SURPASS-ET: Phase III study of ropeginterferon alfa-2b versus anagrelide as second-line therapy in essential thrombocythemia. Future Oncol..

[27] Mesa R., Komatsu N., Gill H., Jin J., Lee S.E., Hou H.A., Sato T., Qin A., Urbanski R., Shih W. (2022). MPN-545 Surpass-ET: Ropeginterferon Alfa-2b (P1101) vs. Anagrelide as Second Line Therapy in Essential Thrombocythemia. Clin. Lymphoma Myeloma Leuk..

[28] Kiladjian J.J., Klade C., Georgiev P., Krochmalczyk D., Gercheva-Kyuchukova L., Egyed M., Dulicek P., Illes A., Pylypenko H., Sivcheva L. (2022). Long-term outcomes of polycythemia vera patients treated with ropeginterferon Alfa-2b. Leukemia.

[29] Morishita S., Komatsu N., Kirito K., Koda A.H., Sekiguchi Y., Tsuneda S., Noda N. (2011). Alternately binding probe competitive PCR as a simple, cost-effective, and accurate quantification method for JAK2V617F allele burden in myeloproliferative neoplasms. Leuk. Res..

[30] Shirane S., Araki M., Morishita S., Edahiro Y., Sunami Y., Hironaka Y., Noguchi M., Koike M., Sato E., Ohsaka A. (2015). Consequences of the JAK2V617F allele burden for the prediction of transformation into myelofibrosis from polycythemia vera and essential thrombocythemia. Int. J. Hematol..

[31] Passamonti F., Rumi E. (2009). Clinical relevance of JAK2 (V617F) mutant allele burden. Haematologica.

[32] Moliterno A.R., Kaizer H., Reeves B.N. (2023). JAK2(V617F) allele burden in polycythemia vera: Burden of proof. Blood.

[33] Morishita S., Takahashi K., Araki M., Hironaka Y., Sunami Y., Edahiro Y., Tsutsui M., Ohsaka A., Tsuneda S., Komatsu N. (2015). Melting curve analysis after T allele enrichment (MelcaTle) as a highly sensitive and reliable method for detecting the JAK2V617F mutation. PLoS ONE.

[34] Boyd E.M., Bench A.J., Goday-Fernandez A., Anand S., Vaghela K.J., Beer P., Scott M.A., Bareford D., Green A.R., Huntly B. (2010). Clinical utility of routine MPL exon 10 analysis in the diagnosis of essential thrombocythaemia and primary myelofibrosis. Br. J. Haematol..

[35] Pietra D., Brisci A., Rumi E., Boggi S., Elena C., Pietrelli A., Bordoni R., Ferrari M., Passamonti F., De Bellis G. (2011). Deep sequencing reveals double mutations in cis of MPL exon 10 in myeloproliferative neoplasms. Haematologica.

[36] Beer P.A., Campbell P.J., Scott L.M., Bench A.J., Erber W.N., Bareford D., Wilkins B.S., Reilly J.T., Hasselbalch H.C., Bowman R. (2008). MPL mutations in myeloproliferative disorders: Analysis of the PT-1 cohort. Blood.

[37] Araki M., Yang Y., Masubuchi N., Hironaka Y., Takei H., Morishita S., Mizukami Y., Kan S., Shirane S., Edahiro Y. (2016). Activation of the thrombopoietin receptor by mutant calreticulin in CALR-mutant myeloproliferative neoplasms. Blood.

[38] Cui L., Moraga I., Lerbs T., Van Neste C., Wilmes S., Tsutsumi N., Trotman-Grant A.C., Gakovic M., Andrews S., Gotlib J. (2021). Tuning MPL signaling to influence hematopoietic stem cell differentiation and inhibit essential thrombocythemia progenitors. Proc. Natl. Acad. Sci. USA.

[39] Yang E., Wang M., Wang Z., Li Y., Wang X., Ming J., Xiao H., Quan R., Liu W., Hu X. (2021). Comparison of the effects between MPL and JAK2V617F on thrombosis and peripheral blood cell counts in patients with essential thrombocythemia: A meta-analysis. Ann. Hematol..

[40] Furuya C., Hashimoto Y., Morishita S., Inano T., Ochiai T., Shirane S., Edahiro Y., Araki M., Ando M., Komatsu N. (2023). MPL gene mutation is a possible risk factor for thrombosis in patients with essential thrombocythemia in Japan. Hematology.

[41] Araki M., Yang Y., Imai M., Mizukami Y., Kihara Y., Sunami Y., Masubuchi N., Edahiro Y., Hironaka Y., Osaga S. (2019). Homomultimerization of mutant calreticulin is a prerequisite for MPL binding and activation. Leukemia.

[42] Masubuchi N., Araki M., Yang Y., Hayashi E., Imai M., Edahiro Y., Hironaka Y., Mizukami Y., Kihara Y., Takei H. (2020). Mutant calreticulin interacts with MPL in the secretion pathway for activation on the cell surface. Leukemia.

[43] Rumi E., Pietra D., Pascutto C., Guglielmelli P., Martinez-Trillos A., Casetti I., Colomer D., Pieri L., Pratcorona M., Rotunno G. (2014). Clinical effect of driver mutations of JAK2, CALR, or MPL in primary myelofibrosis. Blood.

[44] Tefferi A., Lasho T.L., Tischer A., Wassie E.A., Finke C.M., Belachew A.A., Ketterling R.P., Hanson C.A., Pardanani A.D. (2014). The prognostic advantage of calreticulin mutations in myelofibrosis might be confined to type 1 or type 1-like CALR variants. Blood.

[45] Tefferi A., Lasho T.L., Finke C., Belachew A.A., Wassie E.A., Ketterling R.P., Hanson C.A., Pardanani A. (2014). Type 1 vs type 2 calreticulin mutations in primary myelofibrosis: Differences in phenotype and prognostic impact. Leukemia.

[46] Ma W., Kantarjian H., Zhang X., Yeh C.H., Zhang Z.J., Verstovsek S., Albitar M. (2009). Mutation profile of JAK2 transcripts in patients with chronic myeloproliferative neoplasias. J. Mol. Diagn..

[47] Cabagnols X., Favale F., Pasquier F., Messaoudi K., Defour J.P., Ianotto J.C., Marzac C., Le Couedic J.P., Droin N., Chachoua I. (2016). Presence of atypical thrombopoietin receptor (MPL) mutations in triple-negative essential thrombocythemia patients. Blood.

[48] Mori Y., Araki M., Morishita S., Imai M., Edahiro Y., Ito M., Ochiai T., Shirane S., Hashimoto Y., Yasuda H. (2023). Clinical features of acquired erythrocytosis: Low levels of serum erythropoietin in a subset of non-neoplastic erythrocytosis patients. Cancer Med..

[49] Sampieri L., Di Giusto P., Alvarez C. (2019). CREB3 Transcription Factors: ER-Golgi Stress Transducers as Hubs for Cellular Homeostasis. Front. Cell Dev. Biol..

[50] Ibarra J., Elbanna Y.A., Kurylowicz K., Ciboddo M., Greenbaum H.S., Arellano N.S., Rodriguez D., Evers M., Bock-Hughes A., Liu C. (2022). Type I but Not Type II Calreticulin Mutations Activate the IRE1alpha/XBP1 Pathway of the Unfolded Protein Response to Drive Myeloproliferative Neoplasms. Blood Cancer Discov..

[51] Ward A.K., Mellor P., Smith S.E., Kendall S., Just N.A., Vizeacoumar F.S., Sarker S., Phillips Z., Alvi R., Saxena A. (2016). Epigenetic silencing of CREB3L1 by DNA methylation is associated with high-grade metastatic breast cancers with poor prognosis and is prevalent in triple negative breast cancers. Breast Cancer Res..

[52] Denard B., Jiang S., Peng Y., Ye J. (2018). CREB3L1 as a potential biomarker predicting response of triple negative breast cancer to doxorubicin-based chemotherapy. BMC Cancer.

[53] Tefferi A., Guglielmelli P., Lasho T.L., Gangat N., Ketterling R.P., Pardanani A., Vannucchi A.M. (2018). MIPSS70+ Version 2.0: Mutation and Karyotype-Enhanced International Prognostic Scoring System for Primary Myelofibrosis. J. Clin. Oncol..

[54] Tefferi A., Guglielmelli P., Lasho T.L., Coltro G., Finke C.M., Loscocco G.G., Sordi B., Szuber N., Rotunno G., Pacilli A. (2020). Mutation-enhanced international prognostic systems for essential thrombocythaemia and polycythaemia vera. Br. J. Haematol..

[55] Ortmann C.A., Kent D.G., Nangalia J., Silber Y., Wedge D.C., Grinfeld J., Baxter E.J., Massie C.E., Papaemmanuil E., Menon S. (2015). Effect of mutation order on myeloproliferative neoplasms. N. Engl. J. Med..

[56] Inano T., Araki M., Morishita S., Imai M., Yasuda H., Nitta H., Ito M., Edahiro Y., Ochiai T., Misawa K. (2019). JAK2 exon 12 mutation in myelodysplastic/myeloproliferative neoplasm with ring sideroblasts and thrombocytosis: Not an exclusive mutation to polycythaemia vera. Br. J. Haematol..

[57] Tong J., Sun T., Ma S., Zhao Y., Ju M., Gao Y., Zhu P., Tan P., Fu R., Zhang A. (2021). Hematopoietic Stem Cell Heterogeneity Is Linked to the Initiation and Therapeutic Response of Myeloproliferative Neoplasms. Cell Stem Cell.

[58] Muzio G., O’Bray L., Meng-Papaxanthos L., Klatt J., Fischer K., Borgwardt K. (2023). networkGWAS: A network-based approach to discover genetic associations. Bioinformatics.

[59] La Manno G., Soldatov R., Zeisel A., Braun E., Hochgerner H., Petukhov V., Lidschreiber K., Kastriti M.E., Lonnerberg P., Furlan A. (2018). RNA velocity of single cells. Nature.

[60] Lange M., Bergen V., Klein M., Setty M., Reuter B., Bakhti M., Lickert H., Ansari M., Schniering J., Schiller H.B. (2022). CellRank for directed single-cell fate mapping. Nat. Methods.

[61] Kessler M.D., Damask A., O’Keeffe S., Banerjee N., Li D., Watanabe K., Marketta A., Van Meter M., Semrau S., Horowitz J. (2022). Common and rare variant associations with clonal haematopoiesis phenotypes. Nature.

[62] Kar S.P., Quiros P.M., Gu M., Jiang T., Mitchell J., Langdon R., Iyer V., Barcena C., Vijayabaskar M.S., Fabre M.A. (2022). Genome-wide analyses of 200,453 individuals yield new insights into the causes and consequences of clonal hematopoiesis. Nat. Genet..

[63] Genovese G., Kahler A.K., Handsaker R.E., Lindberg J., Rose S.A., Bakhoum S.F., Chambert K., Mick E., Neale B.M., Fromer M. (2014). Clonal hematopoiesis and blood-cancer risk inferred from blood DNA sequence. N. Engl. J. Med..

[64] Jaiswal S., Fontanillas P., Flannick J., Manning A., Grauman P.V., Mar B.G., Lindsley R.C., Mermel C.H., Burtt N., Chavez A. (2014). Age-related clonal hematopoiesis associated with adverse outcomes. N. Engl. J. Med..

[65] Avagyan S., Henninger J.E., Mannherz W.P., Mistry M., Yoon J., Yang S., Weber M.C., Moore J.L., Zon L.I. (2021). Resistance to inflammation underlies enhanced fitness in clonal hematopoiesis. Science.

[66] Nielsen C., Bojesen S.E., Nordestgaard B.G., Kofoed K.F., Birgens H.S. (2014). JAK2V617F somatic mutation in the general population: Myeloproliferative neoplasm development and progression rate. Haematologica.

[67] Van Egeren D., Escabi J., Nguyen M., Liu S., Reilly C.R., Patel S., Kamaz B., Kalyva M., DeAngelo D.J., Galinsky I. (2021). Reconstructing the Lineage Histories and Differentiation Trajectories of Individual Cancer Cells in Myeloproliferative Neoplasms. Cell Stem Cell.

[68] Jaiswal S., Ebert B.L. (2019). Clonal hematopoiesis in human aging and disease. Science.

[69] Jones A.V., Chase A., Silver R.T., Oscier D., Zoi K., Wang Y.L., Cario H., Pahl H.L., Collins A., Reiter A. (2009). JAK2 haplotype is a major risk factor for the development of myeloproliferative neoplasms. Nat. Genet..

[70] Jager R., Harutyunyan A.S., Rumi E., Pietra D., Berg T., Olcaydu D., Houlston R.S., Cazzola M., Kralovics R. (2014). Common germline variation at the TERT locus contributes to familial clustering of myeloproliferative neoplasms. Am. J. Hematol..

[71] Tapper W., Jones A.V., Kralovics R., Harutyunyan A.S., Zoi K., Leung W., Godfrey A.L., Guglielmelli P., Callaway A., Ward D. (2015). Genetic variation at MECOM, TERT, JAK2 and HBS1L-MYB predisposes to myeloproliferative neoplasms. Nat. Commun..

[72] Trifa A.P., Banescu C., Tevet M., Bojan A., Dima D., Urian L., Torok-Vistai T., Popov V.M., Zdrenghea M., Petrov L. (2016). TERT rs2736100 A>C SNP and JAK2 46/1 haplotype significantly contribute to the occurrence of JAK2 V617F and CALR mutated myeloproliferative neoplasms—A multicentric study on 529 patients. Br. J. Haematol..

[73] Harutyunyan A.S., Giambruno R., Krendl C., Stukalov A., Klampfl T., Berg T., Chen D., Milosevic Feenstra J.D., Jager R., Gisslinger B. (2016). Germline RBBP6 mutations in familial myeloproliferative neoplasms. Blood.

[74] Rumi E., Harutyunyan A.S., Pietra D., Feenstra J.D., Cavalloni C., Roncoroni E., Casetti I., Bellini M., Milanesi C., Renna M.C. (2016). LNK mutations in familial myeloproliferative neoplasms. Blood.

[75] Masselli E., Pozzi G., Carubbi C., Vitale M. (2021). The Genetic Makeup of Myeloproliferative Neoplasms: Role of Germline Variants in Defining Disease Risk, Phenotypic Diversity and Outcome. Cells.

[76] Poletto V., Rosti V., Villani L., Catarsi P., Carolei A., Campanelli R., Massa M., Martinetti M., Viarengo G., Malovini A. (2012). A3669G polymorphism of glucocorticoid receptor is a susceptibility allele for primary myelofibrosis and contributes to phenotypic diversity and blast transformation. Blood.

[77] Hodeib H., Hai D.A., Tawfik M.A., Allam A.A., Selim A., Elsawy A.A., Youssef A. (2022). CCL2 rs1024611Gene Polymorphism in Philadelphia-Negative Myeloproliferative Neoplasms. Genes.

[78] Ferrer-Marin F., Arroyo A.B., Bellosillo B., Cuenca E.J., Zamora L., Hernandez-Rivas J.M., Hernandez-Boluda J.C., Fernandez-Rodriguez C., Luno E., Hernandez C.G. (2020). miR-146a rs2431697 identifies myeloproliferative neoplasm patients with higher secondary myelofibrosis progression risk. Leukemia.

[79] Lindgren M., Samuelsson J., Nilsson L., Knutsen H., Ghanima W., Westin J., Johansson P.L., Andreasson B. (2018). Genetic variation in IL28B (IFNL3) and response to interferon-alpha treatment in myeloproliferative neoplasms. Eur. J. Haematol..

[80] Grinfeld J., Nangalia J., Baxter E.J., Wedge D.C., Angelopoulos N., Cantrill R., Godfrey A.L., Papaemmanuil E., Gundem G., MacLean C. (2018). Classification and Personalized Prognosis in Myeloproliferative Neoplasms. N. Engl. J. Med..

