# Supplementary material for: Diagnosis- and Prognosis-Related Gene Alterations in BCR::ABL1-Negative Myeloproliferative Neoplasms
Source: Int J Mol Sci. 2023 Aug 21;24(16):13008. doi: 10.3390/ijms241613008 (PMC10455804; doi:10.3390/ijms241613008)
Supplement: Supplementary file 1 [file ijms-24-13008-s001.zip › ijms-2555672-supplementary.pdf]

Supplementary Table S1. List of reported *JAK2* exon 12 mutations.*

Position in AA	CDS Mutation	AA Mutation	COSMIC ID	Type
521	c.1562C>T	p.P521L	COSM9413096	Substitution - Missense
533	c.1597A>G	p.N533D	COSM1462558	Substitution - Missense
535	c.1605G>C	p.M535I	COSM4385850	Substitution - Missense
536	c.1606_1638dup	p.V536_I546dup	COSM27274	Insertion - In frame
537	c.1609T>A	p.F537I	COSM219800	Substitution - Missense
537	c.1609_1641dup	p.F537_F547dup	COSM33705	Insertion - In frame
537	c.1611_1616del	p.F537_K539delinsL	COSM24437	Deletion - In frame
537	c.?	p.F537I	COSM28699	Substitution - Missense
537	c.?	p.F537L	COSM4385852	Substitution - Missense
537	c.?	p.F537_I540>LV	COSM405280	Complex - Deletion - In frame
537	c.?	p.F537_K539delinsL	COSM29884	Deletion - In frame
538	c.1612C>T	p.H538Y	COSM1462563	Substitution - Missense
538	c.1614_1616del	p.H538_K539delinsQ	COSM1757323	Deletion - In frame
538	c.1614_1616delinsATT	p.H538_K539delinsQL	COSM24438	Substitution - Missense
538	c.1614_1616delinsGTT	p.H538_K539delinsQL	COSM28694	Substitution - Missense
538	c.?	p.H538Q	COSM166358	Substitution - Missense
538	c.?	p.H538_I540>DLS	COSM29883	Complex - compound substitution
538	c.?	p.H538_K539del	COSM29882	Deletion - In frame
538	c.?	p.H538_K539delinsQL	COSM29885	Substitution - Missense
539	c.1614_1616delinsTTT	p.K539L	COSM9413097	Substitution - Missense
539	c.1615A>G	p.K539E	COSM30808	Substitution - Missense
539	c.1615A>T	p.K539*	COSM1757325	Substitution - Nonsense
539	c.1615_1616inv	p.K539L	COSM24439	Substitution - Missense
539	c.1616A>T	p.K539I	COSM219801	Substitution - Missense
539	c.?	p.K539I	COSM4166523	Substitution - Missense
539	c.?	p.K539L	COSM28639	Substitution - Missense
540	c.1619T>C	p.I540T	COSM219805	Substitution - Missense
540	c.1620_1621del	p.I540Mfs*3	COSM219802	Deletion - Frameshift
541	c.1622_1627del	p.R541_E543delinsK	COSM26735	Deletion - In frame
541	c.?	p.R541_E543delinsK	COSM29889	Deletion - In frame
542	c.1624_1629del	p.N542_E543del	COSM24440	Deletion - In frame
542	c.1626_1629del	p.N542Kfs*3	COSM219803	Deletion - Frameshift
542	c.?	p.N542_E543del	COSM29886	Deletion - In frame
543	c.1627_1632del	p.E543_D544del	COSM27090	Deletion - In frame
543	c.?	p.E543_D544del	COSM29481	Deletion - In frame
544	c.1631A>G	p.D544G	COSM30805	Substitution - Missense
544	c.?	p.D544_L545del	COSM29880	Deletion - In frame
545	c.1634T>C	p.L545S	COSM30807	Substitution - Missense
546	c.?	p.I546_F547ins11	COSM405279	Insertion - In frame
547	c.1608_1640dup	p.I546_F547insLFHKIRNEDLI	COSM27275	Insertion - In frame
547	c.?	p.F547_N548ins12	COSM29881	Insertion - In frame
547	c.?	p.F547_N548insHKIRNEDLIL	COSM4385847	Insertion - In frame

* Mutations were extracted from the Catalogue Of Somatic Mutations In Cancer (COSMIC) and the positions of mutations were expressed in accordance with the accession No. NM_004972.3. Sorting was conducted under the following conditions: Tissue; Hematopoietic neoplasm and Polycythemia vera, Somatic status; Confirmed somatic and Variant of unknown origin, and Sample type; Tumor sample. Silent mutations were removed from the list.

Supplementary Table S2. List of reported *CALR* frameshift mutations in ET.*

Position in AA	CDS Mutation	AA Mutation	COSMIC ID	Type
361	c.1080_1143delinsGGAAGAAGACAA	p.Q361Efs*52	COSM6022232	Complex - frameshift
363	c.1088_1089insTTGTC	p.E363Dfs*69	COSM5985671	Insertion - Frameshift
363	c.1088delinsTTTGTC	p.E363Vfs*69	COSM5985668	Complex - frameshift
364	c.1089_1141del	p.E364Gfs*7	COSM5985674	Deletion - Frameshift
364	c.1090_1123del	p.E364Nfs*55	COSM3734989	Deletion - Frameshift
364	c.1090_1141del	p.E364Rfs*49	COSM5885137	Deletion - Frameshift
364	c.1091_1142del	p.E364Gfs*49	COSM1738057	Deletion - Frameshift
364	c.1092_1125del	p.E364Dfs*55	COSM1738333	Deletion - Frameshift
364	c.1092_1140delinsCCA	p.E364Dfs*51	COSM3355743	Complex - frameshift
365	c.1093_1138del	p.Q365Rfs*50	COSM3734990	Deletion - Frameshift
365	c.1094_1139del	p.Q365Rfs*50	COSM1738152	Deletion - Frameshift
366	c.1097_1130del	p.R366Kfs*53	COSM1738357	Deletion - Frameshift
367	c.1099_1135del	p.L367Rfs*51	COSM10127809	Deletion - Frameshift
367	c.1099_1141del	p.L367Rfs*49	COSM5885131	Deletion - Frameshift
367	c.1099_1150del	p.L367Tfs*46	COSM1738055	Deletion - Frameshift
367	c.1099_1153delinsAGG	p.L367Rfs*46	COSM5885139	Complex - frameshift
367	c.1099_1156del	p.L367Rfs*44	COSM5885963	Deletion - Frameshift
367	c.1100_1133del	p.L367Rfs*52	COSM4745937	Deletion - Frameshift
367	c.1100_1134delinsA	p.L367Qfs*52	COSM1738339	Complex - frameshift
367	c.1100_1136delinsGGAGGT	p.L367Rfs*53	COSM5885968	Complex - frameshift
367	c.1100_1145del	p.L367Qfs*48	COSM1738150	Deletion - Frameshift
367	c.1100_1152del	p.L367Qfs*4	COSM9565933	Deletion - Frameshift
367	c.?	p.L367Qfs*?	COSM5574296	Deletion - Frameshift
367	c.?	p.L367Xfs*46	COSM3355748	Deletion - Frameshift
368	c.1101_1134del	p.K368Rfs*51	COSM1738368	Deletion - Frameshift
368	c.1101_1146del	p.K368Rfs*47	COSM5885962	Deletion - Frameshift
368	c.1101_1152del	p.K368Rfs*45	COSM1738334	Deletion - Frameshift
368	c.1101_1161del	p.K368Mfs*42	COSM1738360	Deletion - Frameshift
368	c.1102_1137delinsCA	p.K368Qfs*51	COSM1738157	Complex - frameshift
368	c.1103_1139del	p.K368Rfs*50	COSM5885104	Deletion - Frameshift
368	c.1103_1148del	p.K368Rfs*47	COSM3355766	Deletion - Frameshift
368	c.1103_1154del	p.K368Rfs*45	COSM1738343	Deletion - Frameshift
369	c.1105_1138del	p.E369Rfs*50	COSM1738153	Deletion - Frameshift
369	c.1105_1147delinsAGGAGGCAA	p.E369Rfs*50	COSM5885974	Complex - frameshift
369	c.1105_1156del	p.E369Rfs*44	COSM1738158	Deletion - Frameshift
370	c.1108_1138del	p.E370Rfs*50	COSM5731855	Deletion - Frameshift
370	c.1108_1141del	p.E370Rfs*49	COSM3733223	Deletion - Frameshift
370	c.1108_1177del	p.E370Rfs*37	COSM1738364	Deletion - Frameshift
370	c.1109_1160del	p.E370Vfs*43	COSM6022228	Deletion - Frameshift
371	c.1111_1141del	p.E371Rfs*49	COSM1738349	Deletion - Frameshift
371	c.1111_1142delinsT	p.E371Wfs*49	COSM5731860	Complex - frameshift
371	c.1111_1147del	p.E371Rfs*47	COSM1738335	Deletion - Frameshift
371	c.1111_1150delinsTCA	p.E371Sfs*47	COSM9114826	Complex - frameshift
372	c.1113_1143del	p.E372Qfs*48	COSM10127811	Deletion - Frameshift
372	c.1114_1144del	p.E372Qfs*48	COSM3734991	Deletion - Frameshift
372	c.1115_1139del	p.E372Gfs*50	COSM10127812	Deletion - Frameshift
373	c.1116_1146del	p.D373Rfs*47	COSM2704902	Deletion - Frameshift
373	c.1118_1127del	p.D373Afs*54	COSM10127813	Deletion - Frameshift
373	c.1118_1136del	p.D373Gfs*51	COSM1738159	Deletion - Frameshift
373	c.1118_1145delinsCGTTTA	p.D373Afs*50	COSM1738160	Complex - frameshift
374	c.1120_1125delinsTCTTGTCT	p.K374Sfs*57	COSM5703410	Complex - frameshift
374	c.1120_1126delinsTTCTTGTCTTCTTGTCTT	p.K374Ffs*60	COSM5985670	Complex - frameshift
374	c.1120_1131delinsTGCGT	p.K374Cfs*54	COSM1738344	Complex - frameshift
374	c.1120_1140delinsTCTTGTCT	p.K374Sfs*52	COSM5885136	Complex - frameshift
374	c.1121_1139del	p.K374Rfs*50	COSM1738345	Deletion - Frameshift
374	c.1121_1142del	p.K374Rfs*49	COSM9114827	Deletion - Frameshift
374	c.1121_1148del	p.K374Rfs*47	COSM3734992	Deletion - Frameshift
374	c.1122_1123delinsTTGT	p.K374Nfs*57	COSM5703409	Complex - frameshift
374	c.1122_1125del	p.K374Nfs*55	COSM1738328	Deletion - Frameshift
375	c.1122_1141delinsA	p.K375Rfs*49	COSM1738346	Complex - frameshift
375	c.1122del	p.K375Nfs*55	COSM1738350	Deletion - Frameshift
375	c.1123_1125delinsTGTTT	p.K375Cfs*56	COSM1738351	Complex - frameshift
375	c.1123_1125delinsTTTTGTTT	p.K375Ffs*57	COSM5885965	Complex - frameshift
375	c.1124_1133del	p.K375Rfs*52	COSM1738366	Deletion - Frameshift

375	c.1124_1136del	p.K375Rfs*51	COSM6022233	Deletion - Frameshift
375	c.1124_1142del	p.K375Rfs*49	COSM1738337	Deletion - Frameshift
376	c.1125_1126insTTCTTAGTGCT	p.R376Ffs*58	COSM6022230	Insertion - Frameshift
376	c.1126_1131delinsTGCGT	p.R376Cfs*54	COSM5985672	Complex - frameshift
376	c.1126_1148del	p.R376Gfs*5	COSM6852891	Deletion - Frameshift
376	c.1127_1132delinsTTTGC	p.R376Lfs*54	COSM5885105	Complex - frameshift
376	c.1127_1145del	p.R376Qfs*48	COSM4745934	Deletion - Frameshift
377	c.1125_1146del	p.K377Tfs*46	COSM3734993	Deletion - Frameshift
377	c.1129_1139delinsCTCTGCCTCC	p.K377Lfs*53	COSM5037722	Complex - frameshift
377	c.1129_1139delinsCTCTGTC	p.K377Lfs*52	COSM5037721	Complex - frameshift
377	c.1130_1154delinsTCCATCCTTGTC	p.K377Ifs*49	COSM5885106	Complex - frameshift
378	c.1132_1153del	p.E378Rfs*45	COSM1738329	Deletion - Frameshift
379	c.1135del	p.E379Rfs*51	COSM9285102	Deletion - Frameshift
380	c.1136_1137dup	p.E380Rfs*51	COSM4387491	Insertion - Frameshift
380	c.1139_1140insTC	p.E380Dfs*51	COSM3355767	Insertion - Frameshift
380	c.1139del	p.E380Gfs*50	COSM5885111	Deletion - Frameshift
381	c.1141del	p.E381Rfs*49	COSM1738154	Deletion - Frameshift
381	c.1142_1151delinsCGGCATGTC	p.E381Afs*49	COSM5703411	Complex - frameshift
381	c.1143_1154delinsTCCTTGTC	p.E381Dfs*48	COSM1738362	Complex - frameshift
383	c.1145_1146insGACGC	p.E383Tfs*49	COSM5703408	Insertion - Frameshift
383	c.1147_1151del	p.E383Qfs*4	COSM9180538	Deletion - Frameshift
383	c.1147_1154delinsTGTC	p.E383Cfs*46	COSM5037719	Complex - frameshift
383	c.1148_1149insTCCTTGTC	p.E383Dfs*50	COSM5885135	Insertion - Frameshift
383	c.1148_1154delinsGAC	p.E383Gfs*46	COSM4745936	Complex - frameshift
384	c.1145_1149dup	p.D384Qfs*48	COSM9180539	Insertion - Frameshift
384	c.1150_1151ins52	p.D384fs*?	COSM5715393	Insertion - Frameshift
384	c.1150_1154delinsTGTC	p.D384Cfs*46	COSM6022229	Complex - frameshift
384	c.1151_1154del	p.D384Gfs*45	COSM10127810	Deletion - Frameshift
384	c.1151_1154delinsGCAATTGTC	p.D384Gfs*48	COSM3355746	Complex - frameshift
384	c.1151_1154delinsTTTGTC	p.D384Vfs*47	COSM5037720	Complex - frameshift
385	c.1151_1152insTTGTC	p.K385Cfs*47	COSM9565929	Insertion - Frameshift
385	c.1153_1154delinsTCTTGTC	p.K385Sfs*47	COSM1738365	Complex - frameshift
385	c.1153_1154delinsTGTC	p.K385Cfs*46	COSM1738330	Complex - frameshift
385	c.1153_1154insTCTGT	p.K385Ifs*47	COSM4745935	Insertion - Frameshift
385	c.1154_1155insTTGTC	p.K385Nfs*47	COSM1738056	Insertion - Frameshift
385	c.1154del	p.K385Rfs*45	COSM9180541	Deletion - Frameshift
385	c.1154delinsCTTGTC	p.K385Tfs*47	COSM1738331	Complex - frameshift
385	c.1154delinsGTTGTC	p.K385Sfs*47	COSM9311652	Complex - frameshift
385	c.1154delinsTATGTC	p.K385Ifs*47	COSM5985669	Complex - frameshift
385	c.1154delinsTCTGTC	p.K385Ifs*47	COSM3734994	Complex - frameshift
385	c.1154delinsTGTGTC	p.K385Mfs*47	COSM1738155	Complex - frameshift
385	c.1154delinsTTTATC	p.K385Ifs*47	COSM9311653	Complex - frameshift
385	c.?	p.K385Xfs*47	COSM3355749	Insertion - Frameshift
386	c.1154_1155insATGTC	p.E386Cfs*46	COSM1738355	Insertion - Frameshift
386	c.1155_1156insTGTCG	p.E386Cfs*46	COSM1738332	Insertion - Frameshift
387	c.1153_1157dup	p.D387Rfs*45	COSM5885138	Insertion - Frameshift
388	c.1162del	p.D388Mfs*42	COSM9226088	Deletion - Frameshift
404	c.1211_1217del	p.D404Gfs*24	COSM5574343	Deletion - Frameshift
405	c.1214del	p.E405Gfs*25	COSM5753346	Deletion - Frameshift
408	c.1221del	p.D408Mfs*22	COSM9565932	Deletion - Frameshift

* Mutations were extracted from the Catalogue Of Somatic Mutations In Cancer (COSMIC) and the positions of mutations were expressed in accordance with the accession No. NM_004343.3. Sorting was conducted under the following conditions: Tissue; Hematopoietic neoplasm and Essential thrombocythemia, Somatic status; Confirmed somatic and Variant of unknown origin, and Sample type; Tumor sample. Only frameshift mutations are shown in this list.

Supplementary Table S3. List of reported *CALR* frameshift mutations in MF.*

Position in AA	CDS Mutation	AA Mutation	COSMIC ID	Type
363	c.1088_1089insTTGTC	p.E363Dfs*69	COSM5985671	Insertion - Frameshift
364	c.1090_1123del	p.E364Nfs*55	COSM3734989	Deletion - Frameshift
364	c.1091_1142del	p.E364Gfs*49	COSM1738057	Deletion - Frameshift
365	c.1093_1138del	p.Q365Rfs*50	COSM3734990	Deletion - Frameshift
367	c.1099_1101delinsAC	p.L367Tfs*63	COSM9264988	Complex - frameshift
367	c.1099_1132del	p.L367Rfs*52	COSM1738359	Deletion - Frameshift
367	c.1099_1150del	p.L367Tfs*46	COSM1738055	Deletion - Frameshift
367	c.1100_1145del	p.L367Qfs*48	COSM1738150	Deletion - Frameshift
368	c.1102_1137delinsGA	p.K368Efs*51	COSM3355754	Complex - frameshift
368	c.1103_1136del	p.K368Rfs*51	COSM1738151	Deletion - Frameshift
368	c.1103_1145delinsGGAGGAGGG	p.K368Rfs*51	COSM3355744	Complex - frameshift
368	c.1103_1148del	p.K368Rfs*47	COSM3355766	Deletion - Frameshift
368	c.1103_1154del	p.K368Rfs*45	COSM1738343	Deletion - Frameshift
369	c.1105_1138del	p.E369Rfs*50	COSM1738153	Deletion - Frameshift
369	c.1105_1148delinsAGGAGGCAGT	p.E369Rfs*50	COSM3355745	Complex - frameshift
370	c.1108_1144del	p.E370Qfs*48	COSM1738361	Deletion - Frameshift
370	c.1109_1160del	p.E370Vfs*43	COSM6022228	Deletion - Frameshift
371	c.1111_1141del	p.E371Rfs*49	COSM1738349	Deletion - Frameshift
371	c.1112_1142del	p.E371Gfs*49	COSM5703407	Deletion - Frameshift
373	c.1116del	p.D373Tfs*57	COSM6506477	Deletion - Frameshift
373	c.?	p.D373Xfs*?	COSM9312386	Deletion - Frameshift
374	c.1120_1125delinsTGCGT	p.K374Cfs*56	COSM3355758	Complex - frameshift
374	c.1120_1126delinsTACGTA	p.K374Yfs*56	COSM3355765	Complex - frameshift
374	c.1122_1125del	p.K374Nfs*55	COSM1738328	Deletion - Frameshift
375	c.1122del	p.K375Nfs*55	COSM1738350	Deletion - Frameshift
375	c.1123_1133delinsTGCG	p.K375Cfs*53	COSM9264991	Complex - frameshift
375	c.1123_1144del	p.K375Qfs*48	COSM5703406	Deletion - Frameshift
375	c.1124_1133del	p.K375Rfs*52	COSM1738366	Deletion - Frameshift
375	c.1125del	p.K375Nfs*55	COSM5885245	Deletion - Frameshift
376	c.1127_1129delinsTTTGC	p.R376Lfs*55	COSM1738363	Complex - frameshift
377	c.1129_1135delinsCTTTGCGTA	p.K377Lfs*54	COSM5885110	Complex - frameshift
378	c.?	p.E378Xfs*45	COSM6834816	Deletion - Frameshift
379	c.1135_1147del	p.E379Rfs*47	COSM4745635	Deletion - Frameshift
379	c.1135_1152delinsCCTCCTCTTTGTCT	p.E379Pfs*50	COSM1738352	Complex - frameshift
379	c.1136del	p.E379Gfs*51	COSM9264994	Deletion - Frameshift
379	c.1137_1154delinsCCATCCTTGTC	p.E379Dfs*49	COSM1738353	Complex - frameshift
380	c.1138_1212delinsAGGAGGCAGAGGACAAGGAGGATGATGAGGACAA AGATGAGGATGAGGAGGATGAGGAGGACAAGGAGGAAGATGA	p.E380Rfs*62	COSM5968696	Complex - frameshift
380	c.1139_1140insTC	p.E380Dfs*51	COSM3355767	Insertion - Frameshift
380	c.1139_1215delinsCAAGGAGGATGATGAGGACAAAGATGAGGATGAG GAGGATGAGGAGGACAAGGAGGAAGTTGA	p.E380Afs*57	COSM7338412	Complex - frameshift
380	c.1139del	p.E380Gfs*50	COSM5885111	Deletion - Frameshift
382	c.1144_1220delinsCAGAGGACAAGGAGGATGATGAGGACAAAGATGA GGATGAGGAGGATGAGGAGGACAAGGAGGAAGATG	p.A382Qfs*57	COSM3355735	Complex - frameshift
384	c.1150_1153delinsTTGTCCTCCTCCTGCCTC	p.D384Lfs*51	COSM5885112	Complex - frameshift
384	c.1151_1154delinsGCAATTGTC	p.D384Gfs*48	COSM3355746	Complex - frameshift
384	c.1151_1154delinsTATGTC	p.D384Vfs*47	COSM3355768	Complex - frameshift
385	c.1153_1154delinsTGTC	p.K385Cfs*46	COSM1738330	Complex - frameshift
385	c.1154_1155insTTGTC	p.K385Nfs*47	COSM1738056	Insertion - Frameshift
385	c.1154delinsCTTGTC	p.K385Tfs*47	COSM1738331	Complex - frameshift
385	c.1154delinsTGTGTC	p.K385Mfs*47	COSM1738155	Complex - frameshift
385	c.1154delinsTTTGTC	p.K385Ifs*47	COSM1738356	Complex - frameshift
386	c.1154_1155insATGTC	p.E386Cfs*46	COSM1738355	Insertion - Frameshift
386	c.1155_1156insTGTCG	p.E386Cfs*46	COSM1738332	Insertion - Frameshift
387	c.1157_1158dup	p.D387Rfs*44	COSM9264993	Insertion - Frameshift
387	c.1159_1177del	p.D387Rfs*37	COSM5967353	Deletion - Frameshift
402	c.1204_1252del	p.E402Rfs*12	COSM10202580	Insertion - Frameshift
413	c.1227_1231del	p.A413Gfs*27	COSM9269787	Deletion - Frameshift

* Mutations were extracted from the Catalogue Of Somatic Mutations In Cancer (COSMIC) and the positions of mutations were expressed in accordance with the accession No. NM_004343.3. Sorting was conducted under the following conditions: Tissue; Hematopoietic neoplasm and Myelofibrosis, Somatic status; Confirmed somatic and Variant of unknown origin, and Sample type; Tumor sample. Only frameshift mutations are shown in this list.

Supplementary Table S4. List of non-canonical mutations in driver genes.*

Gene	position in GRCh38	CDS Mutation	AA Mutation	COSMIC ID	disease	Type
JAK2	9:5054790..5054790	c.842G>A	p.G281D	COSM1757320	PV	Substitution - Missense
JAK2	9:5072585..5072585	c.1735C>T	p.L579F	COSM29111	PV	Substitution - Missense
JAK2	9:5073752..5073752	c.1831T>G	p.L611V	COSM51411	PV	Substitution - Missense
JAK2	9:5073753..5073753	c.1832T>C	p.L611S	COSM21361	PV	Substitution - Missense
JAK2	9:5073764..5073764	c.1843G>C	p.V615L	COSM7410093	PV	Substitution - Missense
JAK2	na	c.?	p.V615L	COSM4166522	PV	Substitution - Missense
JAK2	na	c.?	p.C616Y	COSM25862	PV	Substitution - Missense
JAK2	9:5073770..5073770	c.1849G>A	p.V617I	COSM29117	PV	Substitution - Missense
JAK2	9:5073770..5073773	c.1849_1852delinsTTCC	p.V617_C618delinsFR	COSM29821	PV	Substitution - Missense
JAK2	9:5073770..5073773	c.1849_1852delinsTTTC	p.V617_C618delinsFR	COSM27624	PV	Substitution - Missense
JAK2	9:5073773..5073773	c.1852T>C	p.C618R	COSM29118	PV	Substitution - Missense
JAK2	9:5073781..5073781	c.1860C>A	p.D620E	COSM27063	PV	Substitution - Missense
JAK2	na	c.?	p.I724T	COSM5967236	PV	Substitution - Missense
JAK2	9:5089783..5089783	c.2681A>G	p.D894G	COSM327225	PV	Substitution - Missense
JAK2	9:5126715..5126715	c.3323A>G	p.N1108S	COSM33708	PV	Substitution - Missense
JAK2	9:5073770..5073770	c.1849G>A	p.V617I	COSM29117	ET	Substitution - Missense
JAK2	9:5073774..5073774	c.1853G>T	p.C618F	COSM51410	ET	Substitution - Missense
JAK2	9:5077468..5077468	c.1880A>C	p.E627A	COSM7409717	ET	Substitution - Missense
JAK2	9:5078360..5078360	c.2047A>G	p.R683G	COSM29300	ET	Substitution - Missense
JAK2	9:5089701..5089701	c.2599C>T	p.R867W	COSM7409821	ET	Substitution - Missense
JAK2	9:5089702..5089702	c.2600G>A	p.R867Q	COSM303856	ET	Substitution - Missense
JAK2	na	c.?	p.Y317H	COSM6834815	MF	Substitution - Missense
JAK2	9:5073753..5073753	c.1832T>C	p.L611S	COSM21361	MF	Substitution - Missense
JAK2	9:5073769..5073770	c.1848_1849delinsCT	p.V617F	COSM25834	MF	Substitution - Missense
JAK2	9:5081727..5081727	c.2437T>G	p.Y813D	COSM33707	MF	Substitution - Missense
JAK2	9:5081736..5081738	c.2446_2448del	p.L816del	COSM7409990	MF	Deletion - In frame
MPL	1:43337858..43337858	c.10T>C	p.W4R	COSM909746	ET	Substitution - Missense
MPL	na	c.?	p.P70L	COSM5967233	ET	Substitution - Missense
MPL	1:43338685..43338685	c.356C>T	p.T119I	COSM5369881	ET	Substitution - Missense
MPL	1:43339490..43339490	c.611C>T	p.S204F	COSM28996	ET	Substitution - Missense
MPL	1:43339568..43339568	c.689A>G	p.E230G	COSM5369882	ET	Substitution - Missense
MPL	na	c.?	p.Y252H	COSM8630581	ET	Substitution - Missense

MPL	1:43346476..43346476	c.1012G>C	p.E338Q	COSM7409827	ET	Substitution - Missense
MPL	1:43349288..43349289	c.1494_1495insGTGATCGCTCTG	p.L498_H499insVIAL	COSM142847	ET	Insertion - In frame
MPL	1:43349296..43349296	c.1502T>C	p.V501A	COSM86964	ET	Substitution - Missense
MPL	na	c.?	p.V501A	COSM5885103	ET	Substitution - Missense
MPL	1:43349307..43349307	c.1513A>T	p.S505C	COSM86963	ET	Substitution - Missense
MPL	1:43349308..43349308	c.1514G>A	p.S505N	COSM27286	ET	Substitution - Missense
MPL	1:43349319..43349319	c.1525G>T	p.G509C	COSM43211	ET	Substitution - Missense
MPL	na	c.?	p.R514_P518>K	COSM10046171	ET	Complex - deletion inframe
MPL	1:43349336..43349338	c.1542_1544delinsAGC	p.W515A	COSM29009	ET	Substitution - Missense
MPL	1:43349337..43349337	c.1543T>A	p.W515R	COSM29008	ET	Substitution - Missense
MPL	1:43349337..43349338	c.1543_1544delinsGC	p.W515A	COSM27289	ET	Substitution - Missense
MPL	1:43349337..43349340	c.1543_1546delinsAGGG	p.W515_Q516delinsRE	COSM9571856	ET	Substitution - Missense
MPL	na	c.?	p.W515A	COSM41267	ET	Substitution - Missense
MPL	na	c.?	p.W515R	COSM41266	ET	Substitution - Missense
MPL	na	c.?	p.W515S	COSM5885102	ET	Substitution - Missense
MPL	na	c.?	p.W515X	COSM29616	ET	Substitution - Missense
MPL	1:43349340..43349340	c.1546C>G	p.Q516E	COSM7409610	ET	Substitution - Missense
MPL	1:43352635..43352635	c.1771T>G	p.Y591D	COSM28997	ET	Substitution - Missense
MPL	1:43339489..43339489	c.610T>C	p.S204P	COSM26072	MF	Substitution - Missense
MPL	1:43339490..43339490	c.611C>T	p.S204F	COSM28996	MF	Substitution - Missense
MPL	na	c.?	p.S204P	COSM5369880	MF	Substitution - Missense
MPL	1:43349296..43349296	c.1502T>C	p.V501A	COSM86964	MF	Substitution - Missense
MPL	na	c.?	p.V501A	COSM5885103	MF	Substitution - Missense
MPL	1:43349308..43349308	c.1514G>A	p.S505N	COSM27286	MF	Substitution - Missense
MPL	1:43349310..43349310	c.1516G>A	p.A506T	COSM27287	MF	Substitution - Missense
MPL	1:43349323..43349323	c.1529T>C	p.L510P	COSM27288	MF	Substitution - Missense
MPL	1:43349333..43349334	c.1539_1540insCTGAGCTGCCTG	p.L513_R514insLSCL	COSM133104	MF	Insertion - In frame
MPL	1:43349337..43349337	c.1543T>A	p.W515R	COSM29008	MF	Substitution - Missense
MPL	1:43349337..43349337	c.1543T>C	p.W515R	COSM43212	MF	Substitution - Missense
MPL	1:43349337..43349338	c.1543_1544delinsGC	p.W515A	COSM27289	MF	Substitution - Missense
MPL	na	c.?	p.W515A	COSM41267	MF	Substitution - Missense
MPL	na	c.?	p.W515R	COSM41266	MF	Substitution - Missense

MPL	na	c.?	p.W515S	COSM5885102	MF	Substitution - Missense
MPL	na	c.?	p.W515X	COSM29616	MF	Substitution - Missense
MPL	1:43349349..43349349	c.1555G>A	p.A519T	COSM27290	MF	Substitution - Missense
MPL	na	c.?	p.P565L	COSM5982018	MF	Substitution - Missense
MPL	1:43352639..43352639	c.1775G>A	p.R592Q	COSM2170465	MF	Substitution - Missense
MPL	1:43352734..43352734	c.1870C>G	p.H624D	COSM166674	MF	Substitution - Missense
CALR	19:12943753..12943753	c.1094A>T	p.Q365L	COSM5037723	ET	Substitution - Missense
CALR	19:12943758..12943758	c.1099C>A	p.L367I	COSM4385867	ET	Substitution - Missense
CALR	19:12943762..12943762	c.1103A>G	p.K368R	COSM4385870	ET	Substitution - Missense
CALR	19:12943762..12943762	c.1103A>T	p.K368M	COSM3733224	ET	Substitution - Missense
CALR	19:12943767..12943767	c.1108G>T	p.E370*	COSM6506475	ET	Substitution - Nonsense
CALR	19:12943771..12943771	c.1112A>G	p.E371G	COSM4385872	ET	Substitution - Missense
CALR	19:12943772..12943772	c.1113A>C	p.E371D	COSM1738336	ET	Substitution - Missense
CALR	19:12943772..12943819	c.1113_1160del	p.E371_E386del	COSM5037718	ET	Deletion - In frame
CALR	19:12943773..12943773	c.1114G>T	p.E372*	COSM4385869	ET	Substitution - Nonsense
CALR	19:12943783..12943783	c.1124A>G	p.K375R	COSM4385863	ET	Substitution - Missense
CALR	19:12943788..12943799	c.1129_1140del	p.K377_E380del	COSM9180738	ET	Deletion - In frame
CALR	19:12943801..12943801	c.1142A>C	p.E381A	COSM9565930	ET	Substitution - Missense
CALR	19:12943811..12943816	c.1152_1157del	p.D384_K385del	COSM9180540	ET	Deletion - In frame
CALR	19:12943815..12943815	c.1156G>T	p.E386*	COSM4385865	ET	Substitution - Nonsense
CALR	19:12943824..12943824	c.1165G>T	p.E389*	COSM4385871	ET	Substitution - Nonsense
CALR	19:12943874..12943874	c.1215G>T	p.E405D	COSM9565931	ET	Substitution - Missense
CALR	19:12943732..12943732	c.1073A>G	p.K358R	COSM4385861	MF	Substitution - Missense
CALR	19:12943752..12943752	c.1093C>G	p.Q365E	COSM4385858	MF	Substitution - Missense
CALR	19:12943752..12943802	c.1093_1143del	p.Q365_E381del	COSM4385855	MF	Deletion - In frame
CALR	19:12943765..12943765	c.1106A>G	p.E369G	COSM4385864	MF	Substitution - Missense
CALR	19:12943777..12943777	c.1118A>G	p.D373G	COSM4385866	MF	Substitution - Missense
CALR	19:12943779..12943779	c.1120A>C	p.K374Q	COSM6506476	MF	Substitution - Missense
CALR	19:12943780..12943780	c.1121A>G	p.K374R	COSM3720638	MF	Substitution - Missense
CALR	19:12943782..12943782	c.1123A>G	p.K375E	COSM4385859	MF	Substitution - Missense
CALR	19:12943796..12943796	c.1137G>C	p.E379D	COSM3720629	MF	Substitution - Missense
CALR	19:12943801..12943801	c.1142A>G	p.E381G	COSM3720630	MF	Substitution - Missense
CALR	19:12943811..12943811	c.1152C>G	p.D384E	COSM4385857	MF	Substitution - Missense

CALR	19:12943812..12943812	c.1153A>G	p.K385E	COSM9264992	MF	Substitution - Missense
CALR	19:12943820..12943867	c.1161_1208del	p.D387_E402del	COSM5885079	MF	Deletion - In frame
CALR	19:12943834..12943834	c.1175A>G	p.D392G	COSM4385856	MF	Substitution - Missense
CALR	19:12943850..12943858	c.1191_1199del	p.E398_D400del	COSM5715390	MF	Deletion - In frame

* Mutations were extracted from Catalogue Of Somatic Mutations In Cancer (COSMIC) and the positions of mutations were expressed in accordance with the accession Nos. NM_004972.3 for *JAK2*, NM_005373.2 for *MPL*, and NM_004343.3 for *CALR*. Sorting was conducted under the following conditions: Tissue; Hematopoietic neoplasm and Polycythemia vera, Essential thrombocythemia, or Myelofibrosis, Somatic status; Confirmed somatic and Variant of unknown origin, and Sample type; Tumor sample. Silent mutations are removed from the list.
